# Testing the Reliability of ChatGPT Assistance for Surgical Choices in Challenging Glaucoma Cases

**DOI:** 10.3390/jpm15030097

**Published:** 2025-02-28

**Authors:** Matteo Mario Carlà, Gloria Gambini, Federico Giannuzzi, Francesco Boselli, Laura De Luca, Stanislao Rizzo

**Affiliations:** 1 Ophthalmology Department, Fondazione Policlinico Universitario A. Gemelli, IRCCS, 00168 Rome, Italy; gloria.gambini@unicatt.it (G.G.); federico.giannuzzi01@icatt.it (F.G.); francesco.boselli01@icatt.it (F.B.); stanislao.rizzo@unicatt.it (S.R.); 2 Ophthalmology Department, Catholic University “Sacro Cuore”, 00168 Rome, Italy; 3 Ophthalmology Clinic, Department of Biomedical Sciences, University of Messina, 98100 Messina, Italy; dlclra95t61f158n@studenti.unime.it; 4 Consiglio Nazionale delle Ricerche, Istituto di Neuroscienze, 56124 Pisa, Italy

**Keywords:** ChatGPT, large language models (LLM), glaucoma, artificial intelligence (AI), glaucoma surgery

## Abstract

**Background:** This study’s aim is to assess ChatGPT’s capability to analyze detailed case descriptions in glaucomatous patients and suggest the best possible surgical treatment. **Methods:** We conducted a retrospective analysis of 60 medical records of surgical glaucoma cases, divided into “ordinary” cases (*n* = 40) and “challenging” cases (*n* = 20). We entered every case description into ChatGPT-3.5’s interface and inquired “What kind of surgery would you perform?”. The frequency of accurate surgical choices made by ChatGPT, compared to those reported in patients’ files, was reported. Furthermore, we assessed the level of agreement with three senior glaucoma surgeons, asked to analyze the same 60 cases and outline their surgical choices. **Results:** Overall, ChatGPT surgical choices were consistent with those reported in patients’ files in 47/60 cases (78%). When comparing ChatGPT choices with the three glaucoma specialists, levels of agreement were 75%, 70%, and 83%, respectively. In ordinary cases, we did not report any significant differences when comparing ChatGPT answers with those of the three glaucoma specialists, when both of them were matched with patients’ files (*p* > 0.05 for all). ChatGPT’s performances were lower in “challenging” cases: when compared to patients’ files, the accuracy was 13/20 (65%); when compared to glaucoma specialists, the level of agreement was 50%, 40%, and 70%, respectively. **Conclusion:** In ordinary conditions, ChatGPT was able to propose coherent personalized treatment plans, and its performance was comparable to that of skilled glaucoma specialists but showed its limitations in the evaluation of more complex cases.

## 1. Introduction

Globally, glaucoma is a prevalent cause of permanent blindness, with a complex management [1]. The sole modifiable risk factor for slowing glaucoma progression is lowering intraocular pressure (IOP) [2,3]. Current treatment options include pharmacologic topical therapies reducing aqueous humor production or increasing its outflow, lasers targeting the trabecular meshwork and ciliary body, and incisional surgery [4]. Nevertheless, some individuals will continue to lose their visual function even with the best available therapy [5].

Surgical treatment of glaucoma includes a variety of techniques, starting from the gold standard trabeculectomy, to the newly developed microinvasive glaucoma surgery (MIGS) and, in more challenging cases, glaucoma draining devices (GDDs). Nowadays, a personalized approach in surgery, including the search for risk factors for surgical failure, is fundamental to guarantee long-term IOP control. Several risk factors, such as age, race, conjunctival status, and previous medical or surgical treatments, need to be addressed individually for each patient in order to adopt the best technique [6].

Since the first deep convolutional neural networks (CNNs) were introduced in ophthalmology, artificial intelligence (AI) has advanced quickly, [7] with several research showing the efficacy of AI models in treating glaucoma [8,9,10]. Large language models (LLMs) have drawn a lot of attention in ophthalmology lately, especially the Chat Generative Pretrained Transformer (ChatGPT) series created by OpenAI (San Francisco, CA, USA), which has shown promise in comprehending clinical information and generating appropriate replies [11,12]. Based on the abstract link between words (tokens) inside the neural network, GPT models are trained on a textual database and are able to provide replies that are both coherent and contextually relevant [13]. Prior research indicated that GPT-3.5 approaches the 60% passing threshold on the US Medical Licensing Examination (USMLE) with above 50% accuracy. Moreover, more than 90% of AI answers offered insightful answers, exhibiting deductive thinking that can help human learners [14]. Additional features and restrictions of ChatGPT in ophthalmology have been discussed in the literature [15].

This study’s goal is to assess ChatGPT’s capability to analyze detailed case descriptions in glaucomatous patients and to suggest personalized surgical treatment in line with the standard of care. Additionally, we aimed to compare ChatGPT’s responses with glaucoma specialists’ ones, in order to determine the technology’s potential use as an aid in clinical practice.

## 2. Materials and Methods

### 2.1. Case Collection

A retrospective analysis was conducted on the medical records of glaucoma patients who underwent surgical or para-surgical treatments at Policlinico Universitario Agostino Gemelli between May 2019 and August2023. The study received approval from the hospital’s Institutional Review Board (IRB) and was conducted in accordance with the Declaration of Helsinki. Informed consent was waived due to the anonymization of clinical records and the absence of clinical imaging for this analysis.

Out of over 150 cases provided, three independent graders (F.G., F.B., and L.D.L.) selected a total of 60 cases. The cases included all common and uncommon phenotypes of primary or secondary glaucoma: primary open-angle glaucoma, normal-tension glaucoma, primary angle-closure glaucoma, pseudoexfoliation glaucoma, pigment dispersion glaucoma, glaucomatocyclitic crisis glaucoma, aphakic glaucoma, neovascular glaucoma, and uveitic glaucoma. Cases with incomplete medical history or anamnesis, missing pre-operative data or surgical reports on the computer system, or cases undergoing surgery directly from the emergency room were excluded, since a complete case description was difficult to extract.

Successively, we divided those cases into two subgroups:

1. “Ordinary” cases (*n* = 40): Patients with no prior ocular surgery except for cataracts and significant concomitant diseases or ocular conditions. Intraocular pressure management relied only on topical therapy and/or previous laser treatments.
2. “Challenging” cases (*n* = 20): Patients with much more complex medical history, including prior ocular surgeries, such as failed glaucoma surgeries, vitreoretinal surgeries, anterior segment surgeries, or other ocular conditions affecting the homeostasis of the eye. Moreover, cases with concomitant systemic diseases, which may have impacted surgical choices, were included in this subgroup as well.

Descriptions of each case included patient demographics and medical or ocular anamnesis, actual ocular conditions and topical therapy, referred symptoms, and examination findings. All sensitive data regarding patients were accurately removed, and each case description was anonymized. Finally, after removing data regarding the effective surgical treatment, if performed, these data were summarized in a paragraph by a fourth independent grader (M.M.C.) and submitted to the ChatGPT interface.

### 2.2. ChatGPT

**Figure 1:**
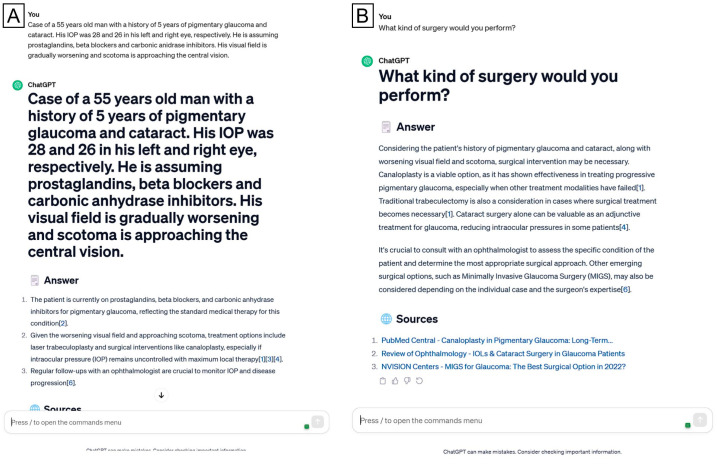
A sample case description, from the “ordinary” cases subgroup, input into the ChatGPT model and corresponding responses.

**Figure 2:**
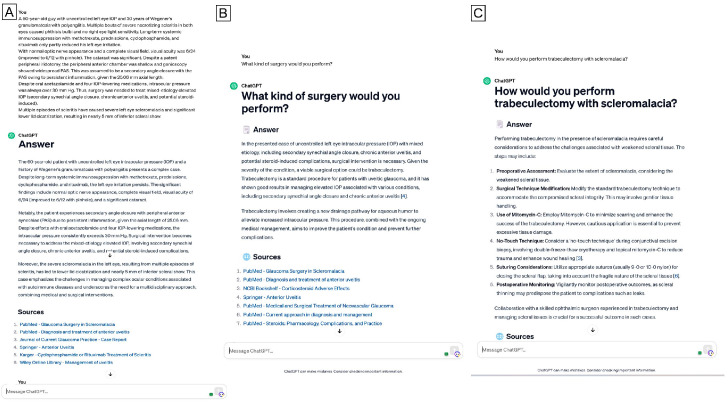
ChatGPT responses in a “challenging” case. Note the capability of producing either coherent customized answers or out-of-context options (“no-touch technique”, “double-freeze-thaw crioterapy”) when asked, “How would you perform trabeculectomy with scleromalacia?”.

**Figure 3:**
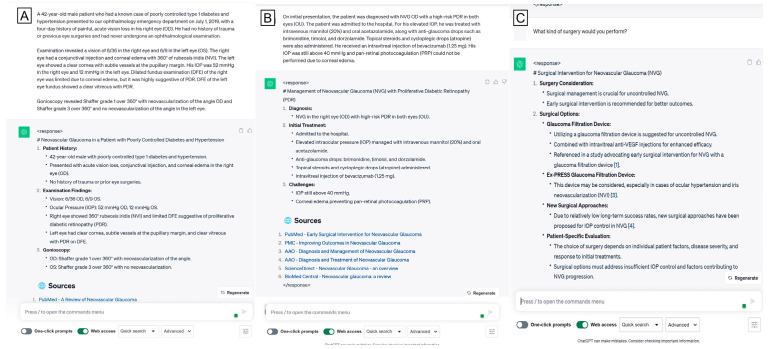
A sample case description from the “challenging” cases subgroup, in which ChatGPT performed a good analysis and offered valid alternatives.

We inputted each case description into the ChatGPT interface (version 3.5). Initially, we instructed the LLM to evaluate the whole case and summarize it in bullet points. Subsequently, we investigated the model’s capacity to provide dependable surgical options. Specifically, we asked, “What type of surgery would you perform?” (Figure 1, Figure 2 and Figure 3). In several cases, we requested ChatGPT for more elucidations of the preferred therapy, predicated on the specific circumstances of the clinical situation. (Figure 2) Specifically, when ChatGPT’s response included a list of several proposed therapies, we inquired, “Among the possible surgical treatments you listed, which one would be your preferred?”. We documented all remarks on our first query on provisional and differential surgical options to facilitate ChatGPT’s learning from previous interactions. The accuracy of surgical decisions made by ChatGPT was compared to the records documented in patients’ files.

Additionally, three senior glaucoma surgeons (G.G., A.S., and S.R.) were asked to analyze the same 30 paragraphs summarizing the clinical cases in a disguised manner and to outline their surgical choices. We then assessed the level of agreement among the three ophthalmology specialists and ChatGPT.

The primary objective of this research was to evaluate the accuracy of ChatGPT’s responses in surgical glaucoma cases, testing its capabilities in both simple and challenging scenarios. As a secondary objective, we aimed to compare ChatGPT’s answers with those of glaucoma specialists facing the same clinical cases.

### 2.3. Statistical Analysis

The statistical analysis was conducted using GraphPad PRISM Software (Version 9.5; GraphPad, La Jolla, CA, USA). To determine the consistency of the answers with patients’ files, ChatGPT’s responses were converted to binary. Chi-square tests, Fisher’s exact tests, and Student’s t-tests were performed where appropriate. The correlation was calculated using Spearman’s coefficient. In all cases, a *p*-value of less than 0.05 was considered statistically significant. Patients and the public were not involved in the design, conduct, reporting, or dissemination plans of the research.

## 3. Results

Glaucoma phenotypes of the 60 selected cases are summarized in Table 1.

ChatGPT’s surgical recommendations aligned with those documented in patients’ files in 47 out of 60 cases (78%). When compared to the assessments of three glaucoma specialists, ChatGPT’s choices were concordant in 45, 42, and 50 cases, corresponding to agreement rates of 75%, 70%, and 83%, respectively. Statistical analysis revealed no significant differences between ChatGPT’s responses and those of the specialists when both were compared to the patients’ files (*p*-values of 0.35, 0.18, and 0.40 for ChatGPT vs. specialist 1, specialist 2, and specialist 3, respectively).

### 3.1. ChatGPT in “Ordinary” Cases

When considering the 40 aforementioned “ordinary” cases, ChatGPT interestingly showed good consistency with patients’ files (33/40 cases, 83% of concordance). Moreover, the levels of agreement with the three specialists were 32/40, 34/40, and 36/40, respectively (80%, 85%, and 90%, respectively).

ChatGPT’s most frequent advice was trabeculectomy (*n* = 21, 53%), followed by glaucoma draining devices (*n* = 7, 18%), MIGS (*n* = 6, 15%), and laser trabeculoplasty (*n* = 3, 7%).

### 3.2. ChatGPT in “Challenging” Cases

The agreements between ChatGPT were lower when focusing on “challenging” cases. When compared to patients’ files, ChatGPT was consistent with 13 out of 20 surgical choices (65%). When compared to glaucoma specialists, the levels of agreement were 10/20 (50%), 8/20 (40%), and 14/20 (70%), respectively. However, even the agreements between the first and second, first and third, and second and third specialists were 14, 15, and 12 (out of 20 cases), respectively.

In challenging conditions, trabeculectomy and glaucoma draining devices (GDDs) were the most frequent suggestion (*n* = 6 for both, 30% each), followed by ab externo cyclophotocoagulation (*n* = 3, 15%) (Supplemental File S1).

**Figure 4:**
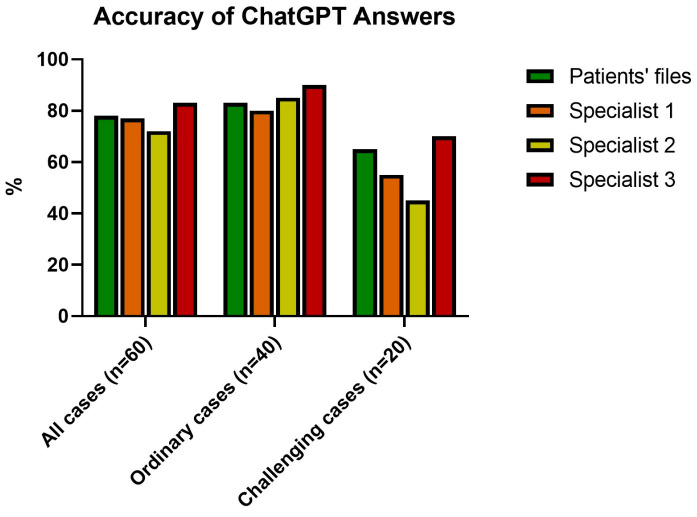
Histogram showing the level of agreement between ChatGPT’s answer and those taken from patients’ files and from the three glaucoma specialists. In “ordinary” cases, there is a strong level of agreement, while “challenging” cases showed much more variability but still a good concordance.

The percentage of agreements between ChatGPT, patients’ files, and glaucoma specialists are summarized in Figure 4.

### 3.3. Errors in Surgical Choices

In a second moment, we focused on those cases in which ChatGPT was not consistent with patients’ files and specialists’ choices. In particular, in the “ordinary” cases subgroup, the discordances of ChatGPT’s answers regarded two cases of primary open-angle glaucoma (POAG), one case of primary angle-closure glaucoma (PACG), and one case of pseudoexfoliation glaucoma. In the “challenging” cases subgroup, ChatGPT showed questionable answers in one case of secondary angle-closure glaucoma combined with scleromalacia, one case of post-traumatic glaucoma, one case of neovascular glaucoma combined with tractional retinal detachment due to branch retinal vein occlusion (BRVO), and one case of inflammatory glaucoma in a case of uveitis–glaucoma–hyphema syndrome.

A summary of discrepancies between ChatGPT responses and those of medical records and of the three specialists for ordinary cases is available in Table 2. The 20 “challenging” glaucoma scenarios are entirely reported in Supplemental File S1, along with ChatGPT and surgeons’ responses.

## 4. Discussion

Investigating the use of LLMs in medicine has garnered a lot of attention in recent months [16,17,18]. Even if they have positive effects across a range of sectors, it is crucial to carefully assess their effectiveness and biases before deciding whether or not they are clinically beneficial [12,19]. In this investigation, we gathered 60 clinical cases to examine ChatGPT’s capacity to define a correct surgical plan for glaucomatous patients. In particular, we divided our sample into “ordinary” and “challenging” cases, to test ChatGPT’s ability to perform an in-depth analysis of a clinical case and to offer personalized treatments in line with the standard of care. Moreover, we tried to assess the level of agreement of this tool with three glaucoma specialists, who were asked to analyze the same pool of clinical cases.

Overall, ChatGPT showed a significant rate of agreement with the surgical procedures employed in patients’ files (78%). Moreover, a good agreement, around 75–80%, was also found when comparing ChatGPT and the three glaucoma specialists. As expected, when “ordinary” clinical cases are submitted to the LLM interface, the accuracy of the analysis rises, reaching 85% concordance with patients’ files. In these cases, ChatGPT is able to offer several possibilities and, if asked, can justify each of them based on the published literature.

When focusing on “challenging” cases, ChatGPT exhibited the capacity to produce customized treatment plans, which were revealed to be much more inaccurate, with the level of agreement lowering to 65%, compared to patients’ files. On the other side, the fact that glaucoma specialists were also unable to define a unique treatment choice suggests that complex cases may require multimodal management and that different kinds of treatments might equally be performed. Notably, the chatbot was able to provide coherent answers among the proposed options, but sometimes it was flanked by some out-of-context techniques (e.g., the “no-touch technique” and “double-freeze-thaw crioterapy” in Figure 2), which significantly hindered the accuracy of the response.

Frequent cases of glaucoma are indeed simpler to manage than uncommon, atypical, and more difficult cases. Even if ChatGPT in those cases showed lower agreement rates, surgical plans seemed generally coherent and relied on “classic” techniques, such as trabeculectomy, GDDs, or cyclophotocoagulation, compared to the tendency of the three surgeons in our research to prefer innovative surgical techniques (such as MIGS) or combined techniques. Overall, we can hypothesize that ChatGPT mainly bases its answers on major reports in the literature, which are focused on techniques that have been available for many years; this approach results in a lesser variety of options, which are, however, the most frequently used and coherently applicable to diverse surgical cases. Nevertheless, in some cases (see Supplemental File S1), ChatGPT lacked a comprehensive assessment and was not able to suggest non-glaucoma surgery or combined surgery (e.g., cataract surgery or vitrectomy) in cases in which all three surgeons agreed on this kind of approach.

In a study conducted by Kianian et al., ChatGPT was able to both create content and rewrite information regarding uveitis, in order to make them easier to digest and aid patients in learning more about this pathology and treat it more adequately [20]. Moreover, according to recent research, ChatGPT accurately diagnosed nine general ophthalmology cases (90%), while Isabel Pro, one of the most popular and accurate diagnostic assistance systems, correctly diagnosed one out of ten instances (10%) [21]. Delsoz et al. recently analyzed the diagnostic capabilities of ChatGPT in a pool of glaucoma patients. Based on 11 clinical reports with primary and secondary glaucoma features, they discovered that ChatGPT was around 72.7% accurate in preliminary diagnoses. They reported three cases of misdiagnosis, regarding atypical glaucoma presentations: glaucomatocyclitic crisis, aqueous misdirection, and inflammatory glaucoma [22]. Furthermore, they showed that, compared to ophthalmology residents, ChatGPT routinely offers a higher number of differential diagnoses [22]. Finally, our group recently demonstrated that ChatGPT 4 outperforms the latest Google LLM, Gemini, when facing either glaucoma or surgical retina clinical cases [23,24].

Utilizing an LLM in a clinical context has the benefit of providing faster, more unbiased, and accurate responses that are also always accessible. Even among highly skilled glaucoma professionals, there is little consensus and great subjectivity in the therapy of glaucoma due to the significant overlap between physiological and pathological criteria [25]. On the other side, in some of the cases we analyzed in our research, ChatGPT was only able to present a list of possible surgical treatments, being more or less relevant when applied to specific clinical cases. As expected, the LLM was not always able to make an arbitrary choice among the proposed treatments, although skilled in specifying the advantages and disadvantages of each of them. Glaucoma specialists were more analytical and made the precise choices that were needed in clinical practice, being also able to examine and analyze visual field printouts and clinical photos, eventually present in the case descriptions. Due to the absence of objective data from diagnostic tests, text-only input may result in more generic differential diagnoses, which may make it more difficult to make precise treatment choices.

Although ChatGPT has demonstrated encouraging results, its immediate application in clinical care settings may be constrained. This is because the incapacity to interpret diagnostic data, such as eye fundus pictures or visual fields, may impair the ability to conduct comprehensive examinations and furnish glaucoma specialists with accurate diagnoses. Given how much ophthalmology depends on visual examination and imaging for patient diagnosis and treatment, only future releases may be able to hone the analysis of medical records [26]. Further, a critical limitation of ChatGPT’s recommendations is its bias toward established surgical techniques, such as trabeculectomy and GDDs, while offering minimal consideration for modern approaches like MIGS. This tendency likely stems from its reliance on major published reports, which often emphasize long-standing, well-documented procedures rather than emerging surgical trends. In current clinical practice, MIGS has gained increasing relevance due to its improved safety profile and faster recovery times, particularly for patients with mild to moderate glaucoma. Future AI iterations should integrate real-time updates from contemporary literature and clinical guidelines to provide more personalized, evidence-based recommendations. Additionally, multimodal AI models capable of analyzing patient-specific factors, such as anterior chamber anatomy and prior surgical history, could significantly enhance decision-making by aligning surgical choices with the latest advancements in glaucoma care.

Our research does have several limitations. First of all, due to its retrospective nature, the case description were based on patients’ clinical records, which could have missed in-depth investigations of clinical conditions. Specifically, a main bias of our research was the arbitrary selection of the information regarding patients’ medical history, which on one side, was necessary to avoid useless data to be analyzed but, on the other side, may have influenced the decision of the surgeons and the chatbot. In order to minimize selection and summarizing biases, the grader who submitted the cases in the chatbot interface did not have access to effective surgical reports. Moreover, we focused on a small number of cases; therefore, future research might examine a larger number of cases to corroborate our findings. In addition, we did not evaluate accuracy using more sophisticated GPT versions, such as GPT 4, which should be more accurate. We indeed chose version 3.5, which is freely accessible and thus much easier to access. Not unique to glaucoma, another fundamental drawback of LLMs such as ChatGPT 3.5 is their incapacity to handle multimodal data, an essential component of medical diagnosis. These shortfalls are on the verge of being overcome by the release of more recent chatbots, which have already shown good diagnostic capabilities facing ophthalmological imaging [27]. Finally, due to the presence of multiple answers, in particular, in challenging cases, the strong simplification required for the purposes of statistical analysis may prove to be a further limitation in the interpretation of our sample data.

In conclusion, LLMs will likely play a significant role in ophthalmic clinical settings, aiding in the development of an even more personalized approach. ChatGPT, in particular, has already gained popularity recently for ophthalmic education but also has good results in the analysis of clinical cases of glaucomatous patients. In ordinary conditions, ChatGPT’s performance was comparable to that of skilled glaucoma specialists, however, showing its limitations in the evaluation of more complex cases.

In the future, especially when multimodal data interpretation is enabled, ChatGPT could become a helpful aid in the schematic analysis of clinical cases, not only in a glaucoma setting, thus offering a list of possible treatments in a rapid, impartial, and accurate way.

## Figures and Tables

**Table 1:** Glaucoma phenotype of the 60 selected medical records.

Glaucoma Phenotype	*n* = 60 (%)
Primary open-angle glaucoma	19 (31.7%)
Primary angle-closure glaucoma	10 (16.7%)
Pigmentary glaucoma	7 (11.7%)
Pseudoexfoliative glaucoma	6 (10.0%)
Neovascular glaucoma	8 (13.3%)
Post-vitrectomy glaucoma	5 (8.3%)
Uveitic glaucoma	2 (3.3%)
Secondary angle-closure glaucoma	2 (3.3%)
Post-traumatic glaucoma	1 (1.7%)

**Table 2:** Comparison of surgical choices in different “ordinary” glaucoma cases between ChatGPT, patients’ medical records, and three specialists: discrepancies in answers.

Case	ChatGPT Answer	Patients’ Files	Specialists’ Answer
Primary open-angle glaucoma (POAG) in a 53-year-old pseudophakic patient with progressive arcuate scotoma in his left eye after two years of latanoprost-timolol-brimonidine topical therapy. Pre-operative IOP was 22 mmHg.	Trabeculectomy	MIGS	S1: MIGS
			S2: MIGS
			S3: Trabeculectomy
Primary angle closure (PACG) in a 64-year-old patient who developed acute primary angle closure 1 month before and was treated with prompt cataract surgery. IOP under triple topical therapy was still 35 mmHg and gonioscopy revealed peripheral anterior synechiae for 120°.	Iridectomy	Trabeculectomy	S1: Trabeculectomy
			S2: Trabeculectomy
			S3: MIGS
A 39-year-old patient with a primary diagnosis of pseudoexfoliation glaucoma (PEXG), visible Sampaolesi line at gonioscopy assessment, and no history of topical therapy. IOP was 32 mmHg.	Trabeculectomy	SLT	S1: SLT
			S2: SLT
			S3: SLT
A 56-year-old patient with POAG in his right eye experiencing progressive visual field loss (mean MD loss 2.1/year), under topical therapy with timolol, tafluprost, and dorzolamide. Anterior segment examination revealed an evident nuclear cataract, and IOP was 30 mmHg.	Trabeculectomy	Cataract surgery + trabeculectomy	S1: Cataract surgery alone
			S2: Cataract surgery + trabeculectomy
			S3: Cataract surgery + trabeculectomy

MIGS = minimally invasive glaucoma surgery; SLT = selective laser trabeculoplasty.

## Data Availability

The data that support the findings of this study are available from the corresponding author, M.M.C., upon reasonable request.

## Supplementary Materials

The following supporting information can be downloaded at: https://www.mdpi.com/article/10.3390/jpm15030097/s1.
