# Supplementary material for: Testing the Reliability of ChatGPT Assistance for Surgical Choices in Challenging Glaucoma Cases
Source: J Pers Med. 2025 Feb 28;15(3):97. doi: 10.3390/jpm15030097 (PMC11943350; doi:10.3390/jpm15030097)
Supplement: Supplementary file 1 [file jpm-15-00097-s001.zip › jpm-3404928-supplementary.pdf]

Challenging glaucoma cases

1. A 65-year-old male presented with a post-traumatic aniridia associated with glaucoma affecting his right eye. The patient suffered a closed globe injury associated with left retinal detachment, which was treated with pars plana vitrectomy (PPV) and silicone oil (SO) tamponade. He was already pseudophakic. His best-corrected visual acuity (BCVA) was counting fingers and intraocular pressure (IOP) was 30 mmHg, without ocular hypotensive agents. Slit-lamp examination showed total iris absence associated with partial (<2 clock h) loss of zonules. Retina was attached with no signs of proliferative vitreoretinopathy. The cup to disk (C/D) ratio was 0.7. Glaucoma eyedrops (dorzolamide-timolol combination), combined with dexamethasone, were started. After SO removal, the IOP gradually increased to 40 mm Hg and became refractive to antiglaucoma medications, with the patient complaining of increasing glare and photophobia.

ChatGPT answer: Trabeculectomy

Medical record: Trabeculectomy

Surgeon 1 plan: GDD

Surgeon 2 plan: GDD

Surgeon 3 plan: Trabeculectomy

2. An 87-year-old female nonsmoker with primary open angle glaucoma, hypertensive retinopathy, and pseudophakia following cataract extraction and intraocular lens (IOL) implant had “cloudy vision” and increased intraocular pressure (IOP). Trabeculectomy had been done in both eyes. Her right eye had 20/50 best corrected visual acuity (BCVA) and her left eye no light perception (NLP). IOP was 14 mmHg on bimatoprost 0.01% right eye one drop daily, dorzolamide/timolol 22.3-6.8 mg/ml twice a day, brimonidine three times a day, pilocarpine 2% three times a day, and methazolamide 25 mg oral tablets twice a day. Anterior segment exam showed flat right and left conjunctival blebs. Bilateral posterior chamber IOLs. Cup-to-disc ratio was 0.9 with progressive cupping and widespread nerve fiber layer loss both eyes. Humphrey's visual field showed a right eye center island of vision with a mean deviation (MD) of –30.

ChatGPT answer: GDD

Medical record: GDD

Surgeon 1 plan: GDD

Surgeon 2 plan: GDD

Surgeon 3 plan: GDD

3. A 42-year-old male patient who had a known case of poorly controlled type 1 diabetes and hypertension presented to our ophthalmology emergency department with a four-day history of painful, acute vision loss in his right eye (OD). He had no history of trauma or previous eye surgeries and had never undergone an ophthalmological examination. Examination revealed a vision of 6/36 in the right eye and 6/9 in the left eye (OS). The right eye had a conjunctival injection and corneal edema with 360° of rubeosis iridis (NVI). The left eye showed a clear cornea with subtle vessels at the pupillary margin. His IOP was 52 mmHg in the right eye and 12 mmHg in the left eye. Dilated fundus examination (DFE) of the right eye was limited due to corneal edema, but it was highly suggestive of proliferative diabetic retinopathy (PDR). DFE of the left eye fundus showed a clear vitreous with PDR. Gonioscopy revealed Shaffer grade 1 over 360° with neovascularization of the angle OD and Shaffer grade 3 over 360° and no neovascularization of the angle in the left eye. His IOP was above 40 mmHg.

ChatGPT answer: Vitrectomy + panretinal photocoagulation

Medical record: Cataract + vitrectomy + panretinal photocoagulation

Surgeon 1 plan: Cataract + vitrectomy + panretinal photocoagulation

Surgeon 2 plan: Cataract + panretinal photocoagulation

Surgeon 3 plan: Cataract + vitrectomy + panretinal photocoagulation

4. Case of a 75 years old female with a 15 years history of primary open angle glaucoma. She assumes beta-blockers, prostaglandins, alpha agonists and oral acetazolamide, but IOP is still uncontrolled (32 in her right eye, 28 in her left eye). Optic nerve shows significant excavation and neuroretinal rhyme reduction. The patient is already pseudophakic and already underwent failed Baerveldt implantation 3 years before in the right eye. Her visual field shows progression in particular in her right eye, with a decrease of -2.2 MD during last year.

ChatGPT answer: Ab-externo cyclophotocoagulation

Medical record: Ab-externo cyclophotocoagulation

Surgeon 1 plan: Ab-externo cyclophotocoagulation

Surgeon 2 plan: GDD exchange

Surgeon 3 plan: Ab-externo cyclophotocoagulation

5. Male patients of 60 years old presented to our clinic with nausea and vomit for the last three hours. Ophthalmic examination showed fixed midriasis, intumescent cataract, mild corneal edema. Intraocular pressure was 55 mmHg in his right eye, which was painful, and 18 in his left eye. He referred previous therapy with topical beta blockers. The patient assume double antiaggregant therapy and anticoagulation.

ChatGPT answer: Peripheral iridotomy

Medical record: Peripheral iridotomy

Surgeon 1 plan: Cataract surgery

Surgeon 2 plan: Cataract surgery

Surgeon 3 plan: Peripheral iridotomy

6. A 60-year-old guy presented with uncontrolled left eye IOP and 30 years of Wegener's granulomatosis with polyangiitis. Multiple bouts of severe necrotizing scleritis in both eyes caused phthisis bulbi and no right eye light sensitivity. Long-term systemic immunosuppression with methotrexate, prednisolone, cyclophosphamide, and rituximab only partly reduced his left eye irritation. With normal optic nerve appearance and a complete visual field, visual acuity was 6/24 with a significant cataract. Despite a patent peripheral iridotomy, the peripheral anterior chamber was shallow and gonioscopy showed widespread PAS. Despite oral acetazolamide and four IOP-lowering medications, intraocular pressure was always over 30 mm. Multiple episodes of scleritis have caused severe left eye scleromalacia and significant lower lid cicatrization, resulting in nearly 5 mm of inferior scleral show.

ChatGPT answer: Trabeculectomy

Medical record: Endoscopic cyclophotocoagulation

Surgeon 1 plan: GDD

Surgeon 2 plan: Endoscopic cyclophotocoagulation

Surgeon 3 plan: GDD

7. In her second trimester of pregnancy, a 26-year-old asthmatic patient who had been treated for juvenile open-angle glaucoma for 12 years with timolol, brimonidine, and latanoprost came to the glaucoma service due to an uncontrolled intraocular pressure (IOP) that her ophthalmologist had noticed was 44 mm Hg in both eyes. She utilized ipratropium and albuterol inhalers on a PRN basis to manage her asthma. The vertical cup/disc ratio was 0.7 in both eyes, and the best-corrected visual acuity was 20/20. The left eye had a shallow inferior arcuate scotoma, whereas the right eye's visual field seemed normal. In the right and left eyes, the central corneal thickness was 550 µm and 557 µm, respectively.

ChatGPT answer: Trabeculectomy

Medical record: Trabeculectomy

Surgeon 1 plan: Goniotomy

Surgeon 2 plan: Goniotomy

Surgeon 3 plan: Trabeculectomy

8. A 57-year-old lady came to our clinic with and uncontrolled POAG in her right more than her left eye with a history of osteoporosis, breast cancer, and osteogenesis imperfecta. Examination of the anterior

segment showed a localized area of thin blue sclera in the temporal quadrant, and examination of the optic nerve indicated glaucomatous cupping with a cup-to-disc ratio of 0.7 in the left eye and 0.9 in the right. Her right eye's IOP was 26 mmHg while she was taking four active principles.

ChatGPT answer: GDD

Medical record: Trabeculectomy

Surgeon 1 plan: GDD

Surgeon 2 plan: GDD

Surgeon 3 plan: GDD

9. Case of a male patient, aged 28 with a diagnosis of Sturge Weber Syndrome, who had previous left trabeculectomy surgery but failed to follow up on appointments, resulting in a subsequent presentation of left-sided exophthalmos and eye discomfort. After presentation with an IOP of 29 mmHg, management with topical latanoprost was able to manage IOP for 6 months, but a relapse at 36 mmHg was then reported.

ChatGPT answer: GDD

Medical record: GDD

Surgeon 1 plan: GDD

Surgeon 2 plan: GDD

Surgeon 3 plan: GDD

10. A patient with a history of five repeat penetrating keratoplasties (PKPs) in his right eye developed high intraocular pressure (IOP) and underwent trabeculectomy surgery. Unfortunately, blebitis developed as an unfavorable consequence. After infection resolution, a scleral patch graft was applied to manage the bleb but the IOP was still 38 mmHg and the eye was painful.

ChatGPT answer: Ab externo cyclophotocoagulation

Medical record: Ab externo cyclophotocoagulation

Surgeon 1 plan: GDD

Surgeon 2 plan: GDD

Surgeon 3 plan: Ab externo cyclophotocoagulation

11. A 42-year-old male patient with right neovascular glaucoma (NVG) secondary to branch retinal vein occlusion presented with iris rubeosis, pseudophakia and a shallow, inferior, tractional retinal detachment. He had already intravitreal bevacizumab injection and IOP was 48 mmHg, with a BCVA of 20/2000.

ChatGPT answer: Trabeculectomy

Medical record: Vitrectomy + photocoagulation

Surgeon 1 plan: Vitrectomy + photocoagulation

Surgeon 2 plan: Vitrectomy + photocoagulation

Surgeon 3 plan: Vitrectomy + photocoagulation

12. Case of secondary open-angle glaucoma in a 36-year-old man, refractory to maximal medical therapy. A meticulous history and careful clinical examination revealed a history of penetrating trauma 15 years ago. Slit lamp examination revealed a self-sealed corneal entry wound, open angles, a retained encapsulated metallic IOFB and siderosis bulbi. Visual acuity was 20/80 and IOP was 27 mmHg.

ChatGPT answer: Vitrectomy.

Medical record: Vitrectomy to remove foreign body

Surgeon 1 plan: Vitrectomy to remove foreign body

Surgeon 2 plan: Vitrectomy to remove foreign body

Surgeon 3 plan: Vitrectomy to remove foreign body

13. A 63-year-old man experienced repeated bouts of symptomatic IOP raise during dialysis sessions with end-stage renal disease. Spikes in intraocular pressure were higher in the right eye, in which peripheral anterior synechiae and angle neovascularization were reported. Intraocular pressure could not be controlled by topical antihypertensive drops. The patient had several co-morbidities, making the medicinal therapeutic options —intravenous glucose, systemic acetazolamide, and mannitol— unsuitable.

ChatGPT answer: GDD

Medical record: GDD

Surgeon 1 plan: GDD

Surgeon 2 plan: GDD

Surgeon 3 plan: GDD

14. A 82-year-old man with nanophthalmos and normal ocular pressure in his right eye underwent uncomplicated phacoemulsification with IOL positioning. After two months, the patient developed malignant glaucoma with an IOP of 31 mmHg. Peripheral iridotomy was not able to relieve the condition.

ChatGPT answer: Trabeculectomy

Medical record: Vitrectomy with hyaloidectomy

Surgeon 1 plan: Vitrectomy with hyaloidectomy

Surgeon 2 plan: Vitrectomy with hyaloidectomy

Surgeon 3 plan: Vitrectomy with hyaloidectomy

15. A 65-year-old man with a history of left ocular trauma and prosthetic iris implantation presented with worsening visual fields, a painful eye and a IOP of 32 mmHg despite maximal medical therapy. The patient had previously undergone pars plana vitrectomy with silicone oil (SO) tamponade and slit lamp examination revealed SO emulsification with hyperoleon in the anterior chamber.

ChatGPT answer: Ab externo cyclophotocoagulation

Medical record: Silicone oil removal

Surgeon 1 plan: Silicone oil removal

Surgeon 2 plan: Silicone oil removal + endoscopic cyclophotocoagulation

Surgeon 3 plan: Silicone oil removal

16. A 20-year-old guy with a history of Marfan syndrome presented with an IOP in his left eye of 35 mmHg. He had an history of bilateral lens subluxation and was left aphakic. Moreover, he developed a retinal detachment in his left eye and underwent vitrectomy, silicone oil (SO) tamponade and successive SO removal. He was under triple topical therapy, but showed worsened BCVA (20/80) and an increasing arcuate scotoma.

ChatGPT answer: GDD

Medical record: GDD

Surgeon 1 plan: GDD

Surgeon 2 plan: GDD

Surgeon 3 plan: GDD

17. A 57-year-old man arrived at our clinic complaining of abrupt blurriness in his right eye that had started approximately a week earlier. He had experienced a cerebrovascular event two years before- The right eye's visual acuity was 20/200, whereas the left eye's was 20/40. A funduscopic examination revealed some firm drusen at the posterior pole in the left eye and submacular hemorrhage and choroidal neovascularization in the right. Four days later the subretinal hemorrhage was worsening in comparison to his earlier image. During subsequent visits, massive subretinal hemorrhage, complete hemorrhagic retinal detachment, vitreous hemorrhage and anterior chamber complete hyphema were reported. IOP at this stage was 50 mmHg.

ChatGPT answer: Vitrectomy

Medical record: Vitrectomy

Surgeon 1 plan: Vitrectomy

Surgeon 2 plan: Vitrectomy

Surgeon 3 plan: Vitrectomy

18. A 52-year-old pseudophakic patient with silicone oil endotamponade for a previous retinal detachment presented with visual loss and intraocular pressure of 60 mm Hg. Slit lamp examination revealed the presence of silicone oil emulsification in the anterior chamber, which was shallower than the fellow eye and an unresponsive pupil, stuck in midriasis

ChatGPT answer: GDD

Medical record: Inferior iridectomy

Surgeon 1 plan: Inferior iridectomy

Surgeon 2 plan: Inferior iridectomy

Surgeon 3 plan: Inferior iridectomy

19. Case of a 54-year-old man who had a uveitic glaucoma in his left eye caused by Fuchs heterochromic iridocyclitis, causing the development of PAS. He was already pseudophakic and experienced central retinal vein occlusion one year ago. He underwent 4 bevacizumab injections and fluorangiography showed peripheral retinal ischemia. Even though the patient was taking topical treatment (timolol 5 mg and bimatoprost 0.3 mg once day), the intraocular pressure was 30 mmHg.

ChatGPT answer: GDD

Medical record: GDD + retinal photocoagulation

Surgeon 1 plan: GDD + Retinal photocoagulation

Surgeon 2 plan: GDD + Retinal photocoagulation

Surgeon 3 plan: GDD + retinal photocoagulation

20. A 72-year-old man had recurrent left eye ocular discomfort and blurred vision. Two years ago, the patient's left eye had a phacoemulsification procedure with the implantation of a posterior chamber IOL. Slit lamp examination showed microhyphema and pseudophakodonesis, while IOL optics substantially covered by the capsulorrhexis edge and no posterior capsule was visible behind the IOL. Under topical therapy medication, intraocular pressure was measured between 20 and 40 mmHg and recurrences were associated with ocular pain.

ChatGPT answer: Trabeculectomy

Medical record: IOL exchange

Surgeon 1 plan: IOL exchange

Surgeon 2 plan: IOL repositioning with optic capture

Surgeon 3 plan: IOL exchange
